# Assessment of the rabbit as a wildlife reservoir of bovine viral diarrhea virus: serological analysis and generation of trans-placentally infected offspring

**DOI:** 10.3389/fmicb.2015.01000

**Published:** 2015-09-23

**Authors:** Dawn M. Grant, Mark P. Dagleish, Claudia Bachofen, Brian Boag, David Deane, Ann Percival, Ruth N. Zadoks, George C. Russell

**Affiliations:** ^1^Vaccines and Diagnostics, Moredun Research Institute, Pentlands Science ParkMidlothian, UK; ^2^The James Hutton InstituteDundee, UK; ^3^Institute of Biodiversity, Animal Health and Comparative Medicine, College of Medical, Veterinary and Life Sciences, University of GlasgowGlasgow, UK

**Keywords:** pestivirus, persistent infection, disease reservoir, wildlife diseases, livestock diseases

## Abstract

Eradication of bovine viral diarrhea virus (BVDV) is ongoing in many European countries and is based on removal of persistently infected (PI) cattle. In this context, low-level risks, including alternative reservoirs of infection, may become more important as the number of BVDV-free herds increases. Alternative reservoirs include livestock, such as sheep and goats, as well as wildlife, including deer and rabbits. Due to the extensive nature of the beef industry in Scotland, where an eradication program started in 2010, contact between cattle and alternative reservoir hosts is common. Seroprevalence to BVDV in rabbit populations can be high. In addition, rabbits can be infected with BVDV by natural routes, indicating that they could be a wildlife reservoir of infection. We analyzed the potential risk to livestock from rabbit populations in the UK by two approaches. First, ∼260 serum samples from free-ranging wild rabbits in Scotland and northern England were tested for BVDV-specific antibodies by ELISA. Only three samples exhibited low level BVDV-specific reactivity, suggesting that BVDV infection of rabbits was not frequent. Second, rabbits were challenged with BVDV at day 7 or 12 of pregnancy. This did not lead to any clinical signs in the infected animals or obvious increases in abortion or stillbirth in the infected dams. Samples from the dams, placental material and ∼130 offspring were tested by BVDV-specific RT-PCR and antibody ELISA. Positive PCR results in the placentas and in the tissues and body fluids of rabbits up to 10 days old showed that trans-placental infection of rabbits with BVDV had occurred. Many of the offspring had BVDV-specific antibodies. These data support the view that a wildlife reservoir of BVDV in rabbit poses a small but non-zero risk of re-infection for BVDV-free cattle herds. Rabbits are susceptible to infection with BVDV but only a small proportion of free-living rabbits in the UK appear to have been infected.

## Introduction

Bovine viral diarrhea (BVD) is an endemic disease, caused by bovine viral diarrhea virus (BVDV), with a significant impact on cattle production and health due to the abortifacient and immunosuppressive effects of infection. Maintenance in the UK herd is driven by persistently infected (PI) animals that were infected *in utero*, so that they tolerate BVDV infection and shed virus continuously.

Bovine viral diarrhea virus PI animals may show no clinical signs as calves, however, they often have a reduced growth rate and productivity and their life-expectancy is significantly reduced. Herds with BVDV generally have reduced reproductive performance and a higher rate of diseases such as diarrhea and pneumonia (Evermann and Faris, 1981) as a consequence of BVDV related immunsuppresion. Because of the economic losses due to BVDV infection, many European countries have undertaken eradication programs. Pioneered by Scandinavian countries, national compulsory eradication programs are ongoing in Austria, Switzerland, Germany, Ireland, and Scotland and are based on detection and removal of PI animals with or without vaccination of uninfected animals in the herd (Stahl and Alenius, 2012). In several other countries, regional and voluntary programs exist.

Scotland has a BVD eradication program based on the identification of PI cattle and the restriction of their sale or movement. However, flaws in the design or implementation of control programs and potential spread from wildlife reservoirs may impact Scotland’s ability to become and remain BVD-free.

Bovine viral diarrhea virus can cross the species barrier relatively easily, particularly into sheep, where it causes a disease clinically indistinguishable from that caused by Border Disease Virus (Carlsson, 1991). Antibodies against BVDV have been detected in a wide range of wild and domesticated ruminant and porcine species (Doyle and Heuschele, 1983; Becher et al., 1997; Scherer et al., 2001; Van Campen et al., 2001) and persistent infection has been demonstrated in sheep, goats, pigs, alpaca, white-tailed deer, eland, mouse deer, and American mountain goats (Terpstra and Wensvoort, 1997; Vilcek et al., 2000; Scherer et al., 2001; Carman et al., 2005; Uttenthal et al., 2005; Passler et al., 2010; Bachofen et al., 2013a). In the early years of BVDV research, a wide range of non-artiodactyls such as horses, cats, dogs, several small laboratory animal species (guinea pig, mouse, rabbit) and embryonated chicken eggs were inoculated with the virus in order to determine the host range (Baker et al., 1954). The only non-artiodactyl animal in which virus could be propagated upon intravenous inoculation was the rabbit. These authors reported (Baker et al., 1954) that calves inoculated with spleen homogenate from rabbits that had been infected with BVDV 5 days earlier showed clinical signs typical of transient BVDV infection. Furthermore, BVDV could be serially passaged, both within rabbits and between rabbits and cattle, using lymphoid cell suspensions (Baker et al., 1954). More recently, a serological survey in Germany showed that 40% of sera sampled from 100 wild rabbits exhibited low neutralizing antibody titres against BVDV (Frolich and Streich, 1998). However, only a third of the positive results could be confirmed by ELISA and no virus could be isolated from any rabbit. A recent experimental study has demonstrated that rabbits can be infected with BVDV by both parenteral and natural routes but shedding of virus was not detected (Bachofen et al., 2014). Thus, there are indications that rabbits could be a natural wildlife reservoir for BVDV. Since rabbits are abundant in countries such as the United Kingdom and Ireland, often living on or near livestock pastures, a BVDV reservoir in rabbits could have significant consequences for BVDV eradication campaigns in these countries, especially toward the end of an eradication scheme. In this study we have used a serological survey of free-ranging wild rabbit populations and experimental infection of pregnant rabbits to determine whether BVDV infected rabbits pose a risk to in-contact livestock.

## Materials and Methods

### Ethics Statement

This study was conducted in the UK in compliance with the Home Office of Great Britain and Northern Ireland ‘Animals (Scientific Procedures) Act 1986’ and with the approval of the Moredun Research Institute Experiments and Ethical Review Committee (E53/14).

### Virus

The BVDV isolate (MRI103) used for the experimental exposures was isolated from the serum of a Scottish PI bovid which was free of maternal antibodies, and passaged six times on bovine turbinate (BT) cells. After three passages, the virus was titrated on BT cells and a multiplicity of infection (MOI) of 0.01 was used for the following passages as previously described (Frolich and Streich, 1998). Medium from the sixth passage, containing BVDV at a titre of 10^6^ TCID50/mL, was clarified by centrifugation at 4000 × *g* for 30 min and stored in aliquots at -80°C before use. All cells, tissue culture medium (Iscove’s modified Dulbecco’s medium, IMDM; Sigma–Aldrich, Dorset, UK) and foetal bovine serum (FBS) used were tested free of pestivirus and antibodies against pestivirus. The 5′UTR and N_pro_ coding region of the isolate were sequenced for phylogenetic typing as previously described (Bachofen et al., 2013b) and MRI103 was determined to be a BVDV-1a virus.

### Animals and Treatments

Twenty mated female New Zealand White rabbits were purchased from a certified breeder with an 80% likelihood of pregnancy, for delivery on estimated day 5 of gestation. The rabbits were acclimatized for 2 days prior to being assigned randomly into two groups of eight animals and one group of four animals that were housed in individual boxes, with each group in a separate room. In cattle, BVDV infection during the first 120 days of pregnancy is thought to result in persistent infection of the fetus (Charleston et al., 2001). Therefore, in challenging pregnant rabbits we used two time points that were within the same portion of the rabbit gestation period (up to day 13). The two groups of eight rabbits were exposed to BVDV intravenously on day 7 (Group 1) or day 12 (Group 2) of gestation via the ear vein with 1ml of virus (10^6^ TCID50) whilst the remaining four rabbits (Group 3) were mock infected with 1 ml of IMDM. A pre-infection blood sample was also collected from each animal. The inoculum of 10^6^ TCID50 had previously been used to induce transient infection in rabbits (Bachofen et al., 2014). One animal from Group 2 and one animal from Group 3 had to be withdrawn from the experiment due to issues with subsequent sampling. The body temperature of each animal was monitored daily by a subcutaneous microchip placed in the neck region (idENTICHIP; Animalcare, York, UK). The animals were observed twice daily until the delivery of the first offspring after which observations were made four times a day. Nesting material was included in all boxes and any live offspring found outside the nest were recovered to it. Any dead offspring or placental tissues found in the boxes were collected and frozen for later analysis. All remaining animals were euthanized at the end of the study (approximately day 10 after birth of the offspring).

At post-mortem examination, samples of lung, heart, liver, spleen, kidney, ileum (sacculus rotundus) and appendix were placed into neutral buffered formal saline, processed routinely through graded alcohols prior to being embedded in paraffin wax and stored at 4°C until required. For detection of BVDV viral RNA, tissue samples were collected into RNAlater (Life Technologies, Paisley, UK). Blood samples were collected post mortem by cardiac puncture and were allowed to clot before drawing off serum, while urine samples were taken directly from the bladder. Samples of serum and urine were frozen under aseptic conditions and stored at -80°C until required.

### RNA Isolation and BVDV Real-Time RT-PCR

RNA isolation from blood or body fluid samples was performed using a viral RNA mini kit (Qiagen, Manchester, UK) according to the manufacturer’s instructions. For tissue samples, homogenisation of about 30 mg of frozen tissue by ceramic beads in RLT buffer (Qiagen) using the Precellys 24 tissue homogenizer was followed by RNA isolation using the RNeasy mini kit (Qiagen).

Buffy coats from blood samples were isolated using a commercial red cell lysis buffer (Promega, Southampton, UK). Subsequent RNA isolation was performed using QIAShredder columns and the RNeasy mini kit (Qiagen).

For simultaneous detection of the viral genome and host beta-actin RNA, an established BVDV-1 specific real time RT-PCR (Willoughby et al., 2006) was used with a generic actin assay (Crook et al., 2012) in a duplex assay on an ABI 7500 sequence detection system (Applied Biosystems-Life Technology, Paisley, UK). Virus-positive RNA samples (Ct < 40) were retested to confirm the result. Additionally, end-point PCR amplification of BVDV RNA directly from serum or urine was performed as described previously (Bachofen et al., 2013b) and the PCR products were characterized by capillary electrophoresis in a commercial instrument (Qiaxcel, Qiagen).

### Sample Collection from Wild Rabbits

**FIGURE 1:**
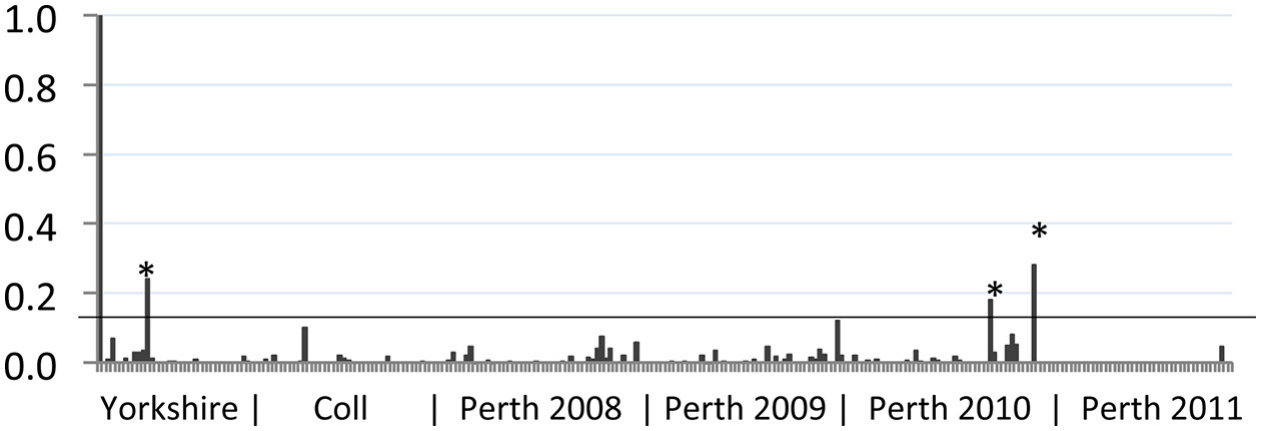
**Reactivity of wild rabbit serum to bovine viral diarrhea (BVD) virus antigen.** The sample to positive (S/P) value (vertical axis) for each sample tested was plotted. Control positive serum (leftmost sample 1) has an S/P value of 1 while control negative serum (sample 2) has a value of 0. The geographic source of samples is indicated beneath the chart, with year of collection for Perth samples. Samples from Yorkshire were collected between 2007 and 2014; samples from Coll were collected between 2008 and 2012. The position of the S/P value cut-off for these samples (0.13) is indicated by a horizontal line. Samples that gave S/P values >0.13 are indicated by asterisks (^∗^).

Serum samples were obtained from wild rabbits shot at three locations in the UK as described previously (Boag et al., 2001, 2013). The majority of samples came from a 400 ha site in Perthshire, Scotland (182; 2008–2011, **Figure 1**), while others were obtained in North Yorkshire, England (31; 2004–2009) and the island of Coll, Scotland (45; 1985–2014). Serum samples were stored at -20°C until required for analysis of serological responses to BVDV antigens.

### ELISA for Detection of BVDV Antibodies

A biphasic, indirect antibody capture ELISA was used to detect BVDV antibodies in serum samples. The test was used essentially as described previously (Bachofen et al., 2014). Briefly, alternate columns of a 96-well ELISA plate (high binding, Greiner Bio-One, Gloucestershire, UK) were coated with antigen from Igepal treated BVDV (isolate C24V) infected cells or with an equivalent antigen preparation from uninfected cells. Prior to usage, plates were blocked for 45 min at room temperature with a solution of 4% milk powder in phosphate buffered saline (PBS) containing 0.05% Tween20 (PBST). The rabbit serum samples were diluted 1:50 in PBST containing 2% milk powder and added in quadruplicates to the plate. After incubation (1 h) and washing, the horseradish peroxidase (HRP) conjugated anti-rabbit Ig antibody was added (P0448; Goat anti-rabbit immunoglobulins/HRP, diluted at 1:1000; Dako UK, Cambridgeshire, UK). Following a further 1 h incubation and a wash step, bound antibody was visualized by adding tetramethylbenzidine substrate (SureBlue, KPL Inc., Gaithersburg, USA). The reaction was stopped after 5 min by addition of 0.18 M sulphuric acid and absorbance at 450 nm was measured in an ELISA plate reader (Dynex MRX_II_, Dynex Technologies, West Sussex, UK). Aliquots of positive terminal serum from BVDV-infected rabbits from a previous experiment were used as positive control, while serum from mock-infected rabbits was used as the negative control (Bachofen et al., 2014). Plate to plate variation was normalized by calculation of the sample to positive (S/P) ratio for each sample relative to the positive and negative control serum on each plate. The following formula was used to calculate S/P values, where the corrected OD is the mean OD of positive antigen wells minus the mean OD of the negative antigen wells inoculated with the same sample:

$$S/P=\frac{\left(corrected\,\;OD\,\;of\,\;sample-corrected\,\;OD\,\;of\,\;negative\,\;control\right)}{\left(corrected\,\;OD\,\;of\,\;positive\,\;control-corrected\,\;OD\,\;of\,\;negative\,\;control\right)}$$

For each ELISA sample set, a cut-off for positive samples was calculated based on all samples (free-ranging rabbits) or on the negative control Group 3 (experimentally infected rabbits) as the arithmetic mean plus 3 standard deviations.

### Histopathology and Immunohistochemistry (IHC)

Paraffin-wax embedded tissue sections were cut (5 μm), mounted on glass microscope slides and stained with haematoxylin and eosin (H&E) prior to evaluation by light microscopy. Selected tissue sections from offspring that were PCR positive for BVDV and for which samples were available were subjected to IHC for BVDV as described previously (Bachofen et al., 2014).

### Statistical Analysis

Analysis of BVDV-specific antibody responses between the two groups of infected rabbits was by Student’s *t*-test (two-tailed, assuming equal variance between the two datasets), while comparisons of infection levels between groups was by Fisher’s exact test (two tailed; Fisher, 1922). Seroprevalence estimates were made using Epitools epidemiological calculators (Sergeant, 2015; http://epitools.ausvet.com.au.) and confidence intervals (CIs) were calculated by the Binomial (Clopper–Pearson) ‘exact’ method within Epitools.

## Results

### Serological Analysis of Wild Rabbit Samples

Rabbit serum samples were tested for BVDV-specific antibodies using an indirect ELISA modified for use with rabbit serum, as described previously (Bachofen et al., 2014). The results of this serosurvey are summarized in **Figure 1**. We have previously shown that rabbits infected by non-parenteral routes developed BVDV-specific antibody responses with S/P values ranging from 0.1 to 1.2 (Bachofen et al., 2014). Among the free-ranging wild rabbit samples tested, the mean S/P value was 0.01 and a cut-off of 0.13 was used to identify potential positive samples. Eleven samples with S/P values greater than 0.1 were retested and of these, four samples remained above 0.1 and three samples had S/P values above 0.13 in both tests. Positive samples originated from Yorkshire (*n* = 1) and Perthshire (*n* = 2). From this analysis the frequency of BVDV-seropositive rabbits in the areas surveyed is estimated at 3.2% (95% CI 0.1–16.7%) for Yorkshire; 0% (95% CI 0.0–7.9%) for Coll; and 1.1% (95% CI 0.1–3.9%) for Perthshire. Although the three sampled areas are geographically distinct, there is no significant difference between regional prevalence estimates and the overall prevalence estimate was 1.2% (95% CI 0.2–3.4%).

### Infection of Pregnant Rabbits

To determine whether rabbits could be infected by BVDV *in utero*, pregnant rabbits were challenged with a BVDV type 1a strain that had previously been shown to induce transient infection of rabbits by parenteral and natural routes (Bachofen et al., 2014). Details of the Groups, litter sizes and survival are given in **Table 1**. One dam in Group 2 did not produce a litter, while three offspring in Group 1, three in Group 2 and one in Group 3 were stillborn. Due to the potential for resorption or abortion of offspring caused by the stress of transport, handling, treatments and sampling, the pregnant dams and their live offspring were not sampled until the end of the experiment (10 days after the birth of the first litters). At this point all surviving animals were euthanized and subject to post-mortem examination. About half of the live-born offspring appeared to be killed by the dams and some were partially eaten (**Table 1**). All dead animals and placentas were removed when they were detected and tissue samples were collected where possible.

**Table 1:** Details of groups and number of animals.

Group (*n*)^a^	Challenged^b^	Litters^c^	Days^d^	Total offspring	Stillborn	Survived^e^
1 (8)	Day 7	8	30–32	67	3	41 (61%)
2 (7)	Day 12	6	32	44	3	18 (41%)
3 (3)	Day 12 (Mock)	3	30–31	20	1	10 (50%)


*^a^Group number and number of animals (n) in each study group from which results were obtained.*

*^b^Day (post-mating) on which challenged. Mock-challenged animals received culture medium only.*

*^c^Number of animals that delivered litters.*

*^d^Day on which litters delivered (days post mating).*

*^e^Number of offspring (and percentage of group total) that survived until the end of the experiment (at least 10 days after birth).*

### Serology

**FIGURE 2:**
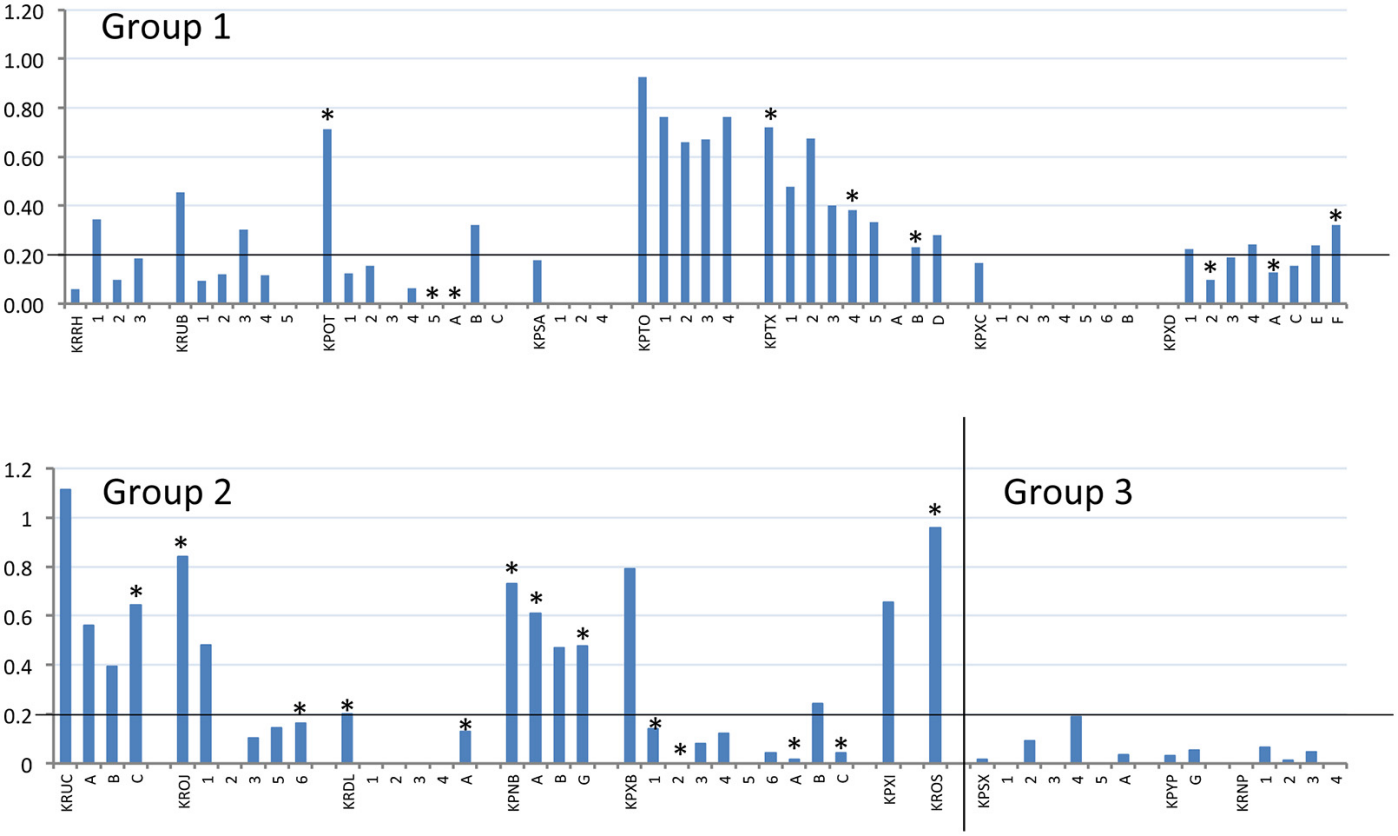
**Reactivity of BVDV-infected rabbits and their offspring to BVDV antigen.** S/P values (vertical axis) of terminal serum samples from all tested dams and offspring (horizontal axis) are plotted. Dams are identified by their four-letter ear-tag codes, while the tested offspring follow the respective dams, identified by single letters or numbers depending on their survival, as described in **Table 2**. Experimental group is indicated within each chart. The position of the S/P value cut-off for these samples (0.2) is indicated by a horizontal line on each chart. All offspring tested here by ELISA were also tested by RT-PCR and positive RT-PCR results are indicated by an asterisk above the relevant column (^∗^).

Terminal serum samples from all dams were tested by BVDV-specific ELISA to demonstrate seroconversion (compared to seronegative pre-infection samples), which was used as indicator of successful infection. All offspring, where a terminal serum sample could be obtained, were tested in the same way to demonstrate presence of BVDV-specific antibodies, without distinction between maternal antibodies or antibodies generated by the offspring. The S/P values of the terminal samples are shown in **Figure 2** and summarized in **Table 2**. A cut-off for samples to be considered positive was calculated from Group 3 as the mean S/P value plus three standard deviations (0.2). The data show that dams in Groups 1 and 2 responded to BVDV infection with a mean S/P value of 0.57. The timing of infection did not have a significant effect on the terminal titre, although the Group 1 dams (infected at day 7 after mating) appeared to have a lower mean S/P value (0.40) than dams in Group 2 (0.75) that were infected 5 days later.

**Table 2:** Outcome of diagnostic analysis.

Animal^a^ (group)	Placenta^b^	Offspring^c^	Offspring BVDV RT-PCR tissue^d^		Offspring BVDV RT-PCR serum (urine)^e^		Serology (offspring)^f^
			POS	NEG	POS	NEG	
KRRH (1)	–	A,B; 1–3	A	B,1,2,3		A,(B),1,2,3	0.06 (+)
KRUB (1)	–	A–C; 1–5	B	1,4,5	(B)	(A),1,2,3,4,5	0.46 (+)
KPOT (1)	29	A,C; 1–7	5	A,1,2,3,4,6	A	C,1,2,3,4,5,6	0.71 (+)
KPSA (1)	–	A-D; 1–6		1,2		A,B,C,1,2,3	0.18 (-)
KPTO (1)	NEG	A,B; 1–4		1,2		B,1,2,3,4	0.93 (+)
KPTX (1)	28	A–D; 1–6	B,4	C,D,1,2,3,5	B	A,(C),D,1,2,3,4,5	0.72 (+)
KPXC (1)	–	A–D; 1–6	A,C	1,2,5,6		B,1,2,3,4,5,6	0.16 (-)
KPXD (1)	–	A,B,C,E,F; 1–4	A,D,F	1,2,3,4	A,F,2	B,C,E,1,3,4	0.00 (+)
							
KRUC (2)	NEG	A–H		A,B	C	A,B,D,(E,G,H)	1.11 (+)
KROJ (2)	31	A; 1–6		1,2,3,4,5,6	6	(A),1,2,3,4,5,6	0.84 (+)
KRDL (2)	26	A; 1–6	A	1,2,3,4	(A)	1,2,3,4	0.20 (-)
KPNB (2)	31	A–G	E,G	A,B,C,D,F	A,(E),G	B,(C,D),F	0.73 (+)
KPXB (2)	NEG	A–C; 1–6	A,C,2	1,3,4,5,6	A,C,1,2	B,3,4,5,6	0.79 (+)
KPXI (2)	–	No litter					0.65 (0)
KROS (2)	29	A–F	B	A,C,D,E,F		(A,C,D,E,F)	0.96 (0)
							
KRNP (3)	–	A,B; 1–4		A,B,3,4		1,2,3,4	0.00 (-)
KPYP (3)	NEG	A-G		A,B		(A,B,C,D,E,F),G	0.03 (-)
KPSX (3)	NEG	A; 1–6		1,2		A,1,2,3,4,5	0.02 (-)

*^a^Animal identifier for each pregnant dam sourced, with experimental group in parenthesis.*

*^b^Detection of BVDV-specific RNA in recovered placentas by real-time RT-PCR. Numbers indicate threshold cycle (Ct) values for detection of BVDV; NEG indicates that no specific amplification was found; – indicates that no tissue was available for analysis.*

*^c^Offspring which died before day 8 are given as letters, while offspring that survived until the end of the experiment are denoted by numbers.*

*^d^Tissue from at least two offspring in each litter were assayed for BVDV-specific RNA by real-time RT-PCR (appendix in all tested except KROS-B and KPOT-A, from which kidney was used). POS indicates detection of BVDV with Ct of 22–25; NEG indicates that no specific amplification was found for the listed offspring.*

*^e^detection of BVDV-specific RNA in serum of offspring by direct end-point RT-PCR or by BVDV-specific real-time RT-PCR. POS indicates detection of BVDV-specific amplicon; NEG indicates that no specific amplification was found for the listed offspring. Where no serum was available, urine was tested by the same method and these results are given in parenthesis. All of the adults were negative for BVDV-specific RT-PCR in serum collected before infection and at port-mortem examination.*

*^f^Bovine viral diarrhea-specific antibody responses were calculated for each animal from a post mortem blood sample (**Figure 2**). The S/P value for each dam is given, while the detection of at least one offspring sample with BVDV-specific antibodies (S/P > 0.2) is indicated by (+). Where all tested offspring were BVDV antibody negative, this is shown as (-). A lack of testable samples is indicated by (0).*

Notably, not all of the dams appeared to respond strongly to infection in challenge Groups 1 and 2. Several had low S/P values (e.g., KPXD, KRRH; **Table 2**), suggesting that the challenge or the immune response may have been sub-optimal in some animals. However, most of these animals had other evidence of infection, such as BVDV RNA detected in placenta or offspring.

### Pathology in Infected Rabbits

No lesions suggestive of BVDV infection were present on examination of H&E sections of any dams or available offspring.

### Immunohistochemistry

Fixed tissues were only available from the dams and those offspring that survived to the end of the experiment (numbered offspring, **Table 2**) and of these, animals KPOT-5, KPTX-4 and KPXB-2 were found to be positive in tissues for BVDV by RT-PCR, whilst additional animals KPXD-2, KROJ-6, and KPXB-1 were RT-PCR positive in serum (**Figure 2**). Sections of all fixed tissues from these six animals only were tested for the presence of pestivirus-specific antigen as described previously (Bachofen et al., 2014). Repeated attempts at IHC gave inconsistent results in the negative control rabbit preparations making interpretation meaningless. Positive and negative controls using BVDV-infected cattle tissue sections gave unequivocal staining patterns, suggesting that the problems were due to the rabbit-specific secondary reagent.

### Detection of Viral RNA

RNA was extracted from placentas recovered from three rabbits in Group 1, six rabbits in Group 2 and two rabbits in Group 3. These samples were assayed for the presence of BVDV-specific RNA by real-time RT-PCR. The results of these assays are summarized in **Table 2** and showed that two animals from Group 1 and four animals from Group 2 shed placentas that contained BVDV RNA. There was no clear association between the detection of BVDV-specific RNA and the terminal BVDV-specific antibody titre in the dams.

Serum and tissues from most offspring were tested for the presence of BVDV RNA by RT-PCR. Where available, serum (or urine) from each animal was tested in a direct end-point PCR assay as described previously (Bachofen et al., 2013b) and these results were repeated where possible by real-time RT-PCR analysis of RNA extracted from serum or urine. Of 117 samples tested, 23 samples were found to be positive (**Table 2**). To confirm these results, RNA was prepared from tissues of at least two offspring from each litter, including all of the offspring found to be potentially positive by direct RT-PCR. RNA was prepared from appendix where available and from kidney in two cases where appendix could not be recovered. These RNA samples were tested by real-time RT-PCR and showed that the majority of RNA samples from tissues of offspring with positively testing serum or urine were also positive by real-time RT-PCR, with Ct values of between 21 and 23; while a small number of real-time RT-PCR positive samples were from tissues of offspring that were BVDV-negative by direct PCR.

It is notable that most of the samples with detectable BVDV RNA came from offspring that were killed by their mothers prior to day 8 after birth. Within the challenged groups, 16 of 52 killed offspring had RT-PCR evidence of BVDV infection while only seven positive samples were found among the 59 offspring that survived to the end of the experiment (*p* < 0.02).

All of the offspring that could be tested for serum antibodies against BVD antigens were also tested by RT-PCR for the presence of viral RNA (**Figure 2**). RT-PCR-negative serum samples had S/P values ranging from zero to 0.76; while RT-PCR-positive samples had S/P values from zero to 0.64. There was no clear correlation between the presence of detectable viral RNA in serum and the antibody response.

## Discussion

The aim of this study was to gather evidence to address the possibility that BVDV-infected rabbits could form a wildlife reservoir and be a risk of re-infection for cattle herds which were free of BVDV and unvaccinated. Serological analysis of more than 250 samples from Yorkshire, Coll and Perth showed that only three samples had S/P values that may represent BVDV-specific antibody responses (**Figure 1**). These samples, however, exhibited high non-specific binding of the negative control antigen in the ELISA and could therefore be false positives. Positive samples could be the result of infection with BVDV, although cross reactivity with other pestiviruses due to antigenic relatedness is also formally possible (Ridpath, 2013). The low frequency of positive samples (1.2%) suggests that BVDV is not established as an endemic infection of rabbits in the UK regions tested and is therefore likely to present a small risk of infection to in-contact livestock.

We have previously demonstrated that rabbits can be productively infected with BVDV type 1a (Bachofen et al., 2014) with virus propagation detected in gut-associated lymphoid tissue and with the development of virus-specific and virus-neutralizing antibody responses. In this study, BVDV viral RNA was detected in rabbits at day 5 after infection but not 3 weeks later, when the animals had seroconverted. To investigate the possibility that infection of pregnant rabbits might lead to the generation of BVDV PI offspring, two groups of rabbits were infected with BVDV1a on day 7 and on day 12 after mating. Following previous results (Bachofen et al., 2014) we would expect the period of potential virus shedding from the dams to have ceased by the end of gestation (23 days after day 7 and 18 days after day 12). Thus the detection of BVDV RNA in the offspring of 80% of infected rabbits is most likely to be the result of trans-placental infection. However the definition of persistent infection by BVDV is based on immune tolerance of the virus and its presence in multiple tissues and body fluids. While BVDV RNA was detected in serum (or urine) of some offspring, the presence of BVDV-specific antibodies in several RT-PCR-positive sera (**Figure 2**) may be the result of maternal transfer or of the immune response by offspring to intra-uterine infection, suggestive of possible transient infection. Further work is required to clarify this.

Of the 15 animals challenged, 14 produced litters, suggesting minimal ill-effects of the transport and infection of the animals. Indeed, the frequency of litters (17 of 18 dams delivered litters with an average litter size of 7.3) was higher than the supplier’s predicted level of 80% and the frequency of stillbirth among the litters was unaffected by group (**Table 1**). However, about half of the offspring (47%) were killed by the dams in the period up to day 8 after birth, after which all remaining offspring survived to the end of the experiment. In Group 1, 39% of the offspring were killed, compared to 59% of Group 2 offspring and 50% of the control group, and this difference was significant between Groups 1 and 2 (*p* < 0.04). This implies that the handling and/or challenge procedures at day 12 of gestation had a significant negative association with survival of the offspring. This appears to be a higher rate of neonatal mortality than in a comparable commercial system (individually housed dams), which showed average litter size of 9.6 and pre-weaning mortality of 15% (Szendro and McNitt, 2012).

It was also notable that the surviving offspring included significantly fewer animals that were RT-PCR positive after testing of serum and tissue samples than the offspring that were found dead (*p* < 0.02). This may suggest that infected offspring are preferentially killed or, alternatively, that transplacental BVDV infection of neonatal rabbits is cleared within the first 10 days after birth, i.e., only in those animals that survived long enough. There was, however, no correlation between the level of BVDV-specific antibodies in the offspring and the detection of viral RNA, suggesting that circulating antibodies in the offspring did not protect them from viraemia, although they may contribute to the resolution of BVDV infection.

It was unclear in this study whether the BVDV-specific antibodies detected in the offspring were the result of maternal transfer or were generated in the offspring following *in utero* infection. Rabbits are immunocompetent at birth but have a restricted antibody repetoire, which continues to diversify up to about 8 weeks of life (Knight and Winstead, 1997). However, it is notable that the two infected dams with the lowest S/P values (KRRH and KPXD; 0 and 0.06, respectively; **Table 2**) delivered offspring with higher S/P values, while all of the other infected dams had higher S/P values than their offspring (**Figure 2**). These distinct patterns suggest that maternal transfer of antibodies may not be the only method by which the offspring gain BVDV-specific antibodies.

The immunohistochemical analysis performed on tissue samples from RT-PCR positive offspring was inconclusive. Greater binding of the labeled secondary antibody to the cytoplasm of epithelia of the appendices and renal medullae was observed in negative control preparations suggesting that goat derived antibodies bound to these rabbit tissues in a non-BVDV antigen mediated manner. Further work is required to optimize these methods for use in rabbits.

The results of this study suggest that while trans-placental infection of BVDV can occur in rabbits, relatively few of the offspring (21%) have evidence of infection from RT-PCR of tissue or body fluids. This supports the serological data that BVDV infection of free-ranging wild rabbits in the UK is infrequent. However, it is not clear from these data whether the infected rabbits were PI in the manner understood for livestock, as this would require further testing at multiple time points. It would also be beneficial to perform longer-term studies on rabbits trans-placentally infected with BVDV to determine whether they shed virus or generate a BVDV-specific antibody response. Although the proportion of infected offspring appears lower in rabbits than in cattle, the proportion of births resulting in infected offspring was high, particularly for challenge at day 12 of gestation, which led to the birth of infected offspring in every litter. This could contribute to maintaining presence of the virus in the rabbit population. The reduced survival of virus-positive offspring compared to virus-negative offspring, however, would limit the opportunity for transmission. The overall seroprevalence of BVDV in rabbits was low but, if the seropositive results represent true positives, 1% of the rabbit population would still translate into a large number of animals, suggesting a non-zero risk of transmission. Furthermore, confidence intervals for prevalence estimates in geographically distinct areas covered a wide range and it is conceivable that seroprevalence may be high in specific areas, as previously suggested by Frolich and Streich (1998) based on field studies in Germany. In summary, there is a non-zero risk of BVDV infection in rabbits and although this is unlikely to be of epidemiological relevance for most control scenarios, it may theoretically play a role in the tail end of an eradication campaign, particularly in agricultural systems with a high likelihood of contact between cattle and rabbits.

## Conflict of Interest Statement

The authors declare that the research was conducted in the absence of any commercial or financial relationships that could be construed as a potential conflict of interest.

## References

[B1] Bachofen C. Grant D. M. Willoughby K. Zadoks R. N. Dagleish M. P. Russell G. C. (2014). Experimental infection of rabbits with bovine viral diarrhoea virus by a natural route of exposure. *Vet. Res.* 45 34 10.1186/1297-9716-45-34 PMC4234416 24690167

[B2] Bachofen C. Vogt H. Stalder H. Mathys T. Zanoni R. Hilbe M. (2013a). Persistent infections after natural transmission of bovine viral diarrhoea virus from cattle to goats and among goats. *Vet. Res.* 44 15 10.1186/1297-9716-44-32 PMC3660168 23675947

[B3] Bachofen C. Willoughby K. Zadoks R. Burr P. Mellor D. Russell G. C. (2013b). Direct RT-PCR from serum enables fast and cost-effective phylogenetic analysis of bovine viral diarrhoea virus. *J. Vir. Meth.* 190 1–3. 10.1016/j.jviromet.2013.03.015 23541784

[B4] Baker J. A. York C. J. Gillespie J. H. Mitchell G. B. (1954). Virus Diarrhea in Cattle. *Am. J. Vet. Res.* 15 525–531. 13207572

[B5] Becher P. Orlich M. Shannon A. D. Horner G. Konig M. Thiel H. J. (1997). Phylogenetic analysis of pestiviruses from domestic and wild ruminants. *J. Gen. Virol.* 78 1357–1366. 10.1099/0022-1317-78-6-1357 9191930

[B6] Boag B. Hernandez A. D. Cattadori I. M. (2013). Observations on the epidemiology and interactions between myxomatosis, coccidiosis and helminth parasites in a wild rabbit population in Scotland. *Eur. J. Wildl. Res.* 59 557–562. 10.1007/s10344-013-0704-0

[B7] Boag B. Lello J. Fenton A. Tompkins D. M. Hudson P. J. (2001). Patterns of parasite aggregation in the wild European rabbit (*Oryctolagus cuniculus*). *Int. J. Parasitol.* 31 1421–1428. 10.1016/S0020-7519(01)00270-3 11595228

[B8] Carlsson U. (1991). Border disease in sheep caused by transmission of virus from cattle persistently infected with bovine virus diarrhea virus. *Vet. Rec.* 128 145–147. 10.1136/vr.128.7.145 1851350

[B9] Carman S. Carr N. Delay J. Baxi M. Deregt D. Hazlett M. (2005). Bovine viral diarrhea virus in alpaca: abortion and persistent infection. *J. Vet. Diag. Invest.* 17 589–593. 10.1177/104063870501700613 16475521

[B10] Charleston B. Fray M. D. Baigent S. Carr B. V. Morrison W. I. (2001). Establishment of persistent infection with non-cytopathic bovine viral diarrhoea virus in cattle is associated with a failure to induce type I interferon. *J. Gen. Virol.* 82 1893–1897. 10.1099/0022-1317-82-8-1893 11457995

[B11] Crook T. Benavides J. Russell G. Gilray J. Maley M. Willoughby K. (2012). Bovine herpesvirus 1 abortion: current prevalence in Scotland and evidence of haematogenous spread within the foetus in natural cases. *J. Vet. Diag. Invest.* 24 662–670. 10.1177/1040638712448187 22649159

[B12] Doyle L. G. Heuschele W. P. (1983). Bovine viral diarrhea virus-infection in captive exotic ruminants. *J. Am. Vet. Med. Assoc.* 183 1257–1259. 6315662

[B13] Evermann J. F. Faris M. A. (1981). Current clinical aspects of bovine viral diarrhea virus-infection. *Bovine Practice* 2 39–41. 10.1002/rmv.677

[B14] Fisher R. A. (1922). On the interpretation of X2 from contingency tables, and the calculation of P. *J. R. Stat. Soc.* 85 87–94. 10.2307/2340521

[B15] Frolich K. Streich W. J. (1998). Serologic evidence of bovine viral diarrhea virus in free-ranging rabbits from Germany. *J. Wildl. Dis.* 34 173–178. 10.7589/0090-3558-34.1.173 9476243

[B16] Knight K. L. Winstead C. R. (1997). Generation of antibody diversity in rabbits. *Curr. Opin. Immunol.* 9 228–232. 10.1016/S0952-7915(97)80140-9 9099798

[B17] Passler T. Ditchkoff S. S. Givens M. D. Brock K. V. DeYoung R. W. Walz P. H. (2010). Transmission of bovine viral diarrhea virus among white-tailed deer (*Odocoileus virginianus*). *Vet. Res.* 41 20. 10.1051/vetres/2009068 PMC2797653 19922743

[B18] Ridpath J. F. (2013). A need to define characteristics to be used in the taxonomy of the expanding pestivirus genus. *Berl. Munch. Tierarztl. Wochenschr.* 126 462–467. 24511820 10.2376/0005-9366-126-462

[B19] Scherer C. F. C. Flores E. F. Weiblen R. Caron L. Irigoyen L. F. Neves J. P. (2001). Experimental infection of pregnant ewes with bovine viral diarrhea virus type-2 (BVDV-2): effects on the pregnancy and fetus. *Vet. Microbiol.* 79 285–299. 10.1016/S0378-1135(00)00357-6 11267789

[B20] Sergeant E. S. G. (2015). *Epitools Epidemiological Calculators. AusVet Animal Health Services and Australian Biosecurity Cooperative Research Centre for Emerging Infectious Disease.* Available at: http://epitools.ausvet.com.au/

[B21] Stahl K. Alenius S. (2012). BVDV control and eradication in Europe -an update. *Jap. J. Vet. Res.* 60 S31–S39. 22458198

[B22] Szendro Z. S. McNitt J. I. (2012). Housing of rabbit does: Group and individual systems: a review. *Livestock Sci.* 150 1–10. 10.1016/j.livsci.2012.09.01

[B23] Terpstra C. Wensvoort G. (1997). A congenital persistent infection of bovine virus diarrhoea virus in pigs: clinical, virological and immunological observations. *Vet. Quart.* 19 97–101. 10.1080/01652176.1997.9694750 9323848

[B24] Uttenthal A. Grondahl C. Hoyer M. J. Houe H. van Maanen C. Rasmussen T. B. (2005). Persistent BVDV infection in mousedeer infects calves - Do we know the reservoirs for BVDV? *Prev. Vet. Med.* 72 87–91. 10.1016/j.prevetmed.2005.08.006 16213611

[B25] Van Campen H. Ridpath J. Williams E. Cavender J. Edwards J. Smith S. (2001). Isolation of bovine viral diarrhea virus from a free-ranging mule deer in Wyoming. *J. Wildl. Dis.* 37 306–311. 10.7589/0090-3558-37.2.306 11310881

[B26] Vilcek S. Paton D. J. Rowe L. W. Anderson E. C. (2000). Typing of pestiviruses from Eland in Zimbabwe. *J. Wildl. Dis.* 36 165–168. 10.7589/0090-3558-36.1.165 10682761

[B27] Willoughby K. Valdazo-Gonzalez B. Maley A. Gilray J. Nettleton P. (2006). Development of a real time RT-PCR to detect and type ovine pestiviruses. *J. Virol. Methods* 132 187–194. 10.1016/j.jviromet.2005.10.007 16309752

